# Antibodies Inhibiting the Type III Secretion System of Gram-Negative Pathogenic Bacteria

**DOI:** 10.3390/antib9030035

**Published:** 2020-07-27

**Authors:** Julia A. Hotinger, Aaron E. May

**Affiliations:** Department of Medicinal Chemistry, School of Pharmacy, Virginia Commonwealth University, Richmond, VA 23219, USA; hotingerja@vcu.edu

**Keywords:** type III secretion system, antibodies, prophylaxis, antibacterials, antibiotics

## Abstract

Pathogenic bacteria are a global health threat, with over 2 million infections caused by Gram-negative bacteria every year in the United States. This problem is exacerbated by the increase in resistance to common antibiotics that are routinely used to treat these infections, creating an urgent need for innovative ways to treat and prevent virulence caused by these pathogens. Many Gram-negative pathogenic bacteria use a type III secretion system (T3SS) to inject toxins and other effector proteins directly into host cells. The T3SS has become a popular anti-virulence target because it is required for pathogenesis and knockouts have attenuated virulence. It is also not required for survival, which should result in less selective pressure for resistance formation against T3SS inhibitors. In this review, we will highlight selected examples of direct antibody immunizations and the use of antibodies in immunotherapy treatments that target the bacterial T3SS. These examples include antibodies targeting the T3SS of *Pseudomonas aeruginosa*, *Yersinia pestis*, *Escherichia coli*, *Salmonella enterica*, *Shigella* spp., and *Chlamydia trachomatis*.

## 1. Introduction

**Figure 1:**
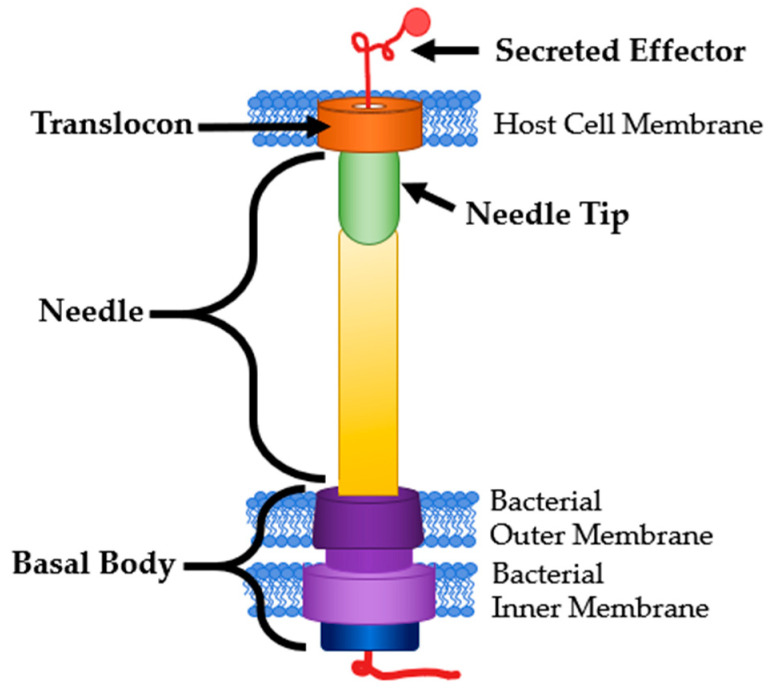
T3SS Structure and Common Targets. Modified from [3].

The type III secretion system (T3SS) is a multimeric protein complex used by many pathogenic Gram-negative bacteria to cause and maintain an infection [1]. Pathogens that use a T3SS include *Chlamydia trachomatis*, *Escherichia coli*, *Pseudomonas aeruginosa*, *Salmonella enterica*, *Shigella* spp., *Vibrio cholerae*, and *Yersinia pestis* [2]. The T3SS functions as a molecular syringe, sometimes called an injectisome that bacteria use to translocate effector proteins directly into a host cell (Figure 1) [3]. The T3SS is comprised of three major components. First, a basal body that anchors the structure to the bacterial membrane containing an ATPase at the base that powers the secretion of proteins. Next, the needle itself acts as a tunnel that spans the extracellular space between the pathogen and host cell. Finally, there is a translocon that forms a pore in the host cell membrane [4]. Due to the small diameter of the needle, the effector proteins must be unfolded to be translocated and then are re-folded after entering the host cell [5]. These effector proteins are responsible for modifying the host cell functions in ways that are beneficial to the pathogen. This includes mechanisms such as reprogramming host machinery to allow for colonization through interference with actin and tubulin, gene expression, or cell cycle progression (*Salmonella* spp., *Shigella* spp.) [6,7]. Some pathogens even interfere with or induce programmed cell death (*Yersinia* spp., *Pseudomonas* spp.) [8,9].

The T3SS is becoming an important anti-virulence target for many reasons. The T3SS is specific to Gram-negative pathogens, meaning any interventions targeting it should not affect commensal bacteria [10]. Bacteria containing a nonfunctional T3SS also have attenuated virulence but are still capable of growth [11,12,13,14,15]. This lends to the theory that inhibiting the T3SS will reduce the selective pressure on the bacterial pathogen to form resistance, leading to slower formation of resistance to T3SS inhibitors [16]. Small molecule inhibitors of the T3SS have been shown to increase survival rates after infection with otherwise lethal doses of bacterial pathogens [10,17].

Mammalian immune systems produce antibodies (Ab) against T3SS proteins when natural infection occurs [18,19,20,21,22]. Due to the high prevalence of infection caused by bacteria utilizing the T3SS, the majority of humans have antibodies to the T3SS of some pathogens already in their system [22]. Durand et al. tested human colostrum samples for Abs against T3SS proteins for *Salmonella* spp., *Shigella* spp., and *E. coli* including the needle tip, translocon, and secreted effectors. They found that every sample collected contained Abs to at least one of the aforementioned proteins and 10% of the samples contained Abs to all 11 proteins tested [22]. When pregnant cattle were vaccinated against *E. coli* with two recombinant T3SS-related proteins, EspB, and γ-intimin, the Abs produced against these antigens was passed to their calves through breast milk [23]. Rabinovitz et al. showed that calves with vaccinated mothers showed markedly higher survival rates after a challenge of enterohemorrhagic *E. coli* (EHEC) than those with sham-vaccinated mothers [24].

Antibody recognition can lead to rapid and robust responses by the immune system, removing the pathogen before any symptoms can be felt or seen in the host. When this is the case, we consider the host to be immune to the pathogen [25]. The presence of anti-T3SS Abs is enough to identify that an individual has come into contact with the pathogen with the T3SS protein in question, but not necessarily that they have immunity. This is because not all Abs have the same immunoprotective properties [26]. Notwithstanding this fact, the presence of the Abs targeting the T3SS and its effectors across multiple bacterial species implies a significant therapeutic potential. The most T3SS structural components are not expressed by non-pathogenic bacteria, allowing for the potential for enhanced specificity. In this review, we will cover selected examples of promising and effective antibody-based treatments and prophylactics that target the T3SS of pathogenic bacteria. 

## 2. Antibody Structure and Function

**Figure 2:**
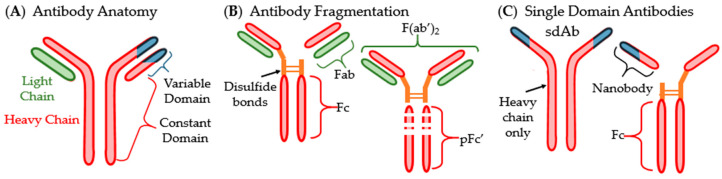
Structure of antibody and common fragmentation types: (**A**) generic anatomy of an antibody; (**B**) visualization of fragmented antibodies; (**C**) single domain antibodies (sdAbs) and their fragmentation.

The majority of antibodies are “Y” shaped immunoglobulin (Ig) proteins that are used by the immune system to recognize antigens. They contain a variable domain on the tips on the Y and bind to antigens. A non-variable or constant domain on the stem of the Y binds to cellular receptors (Figure 2A) [25]. There are five main isotypes of Abs found in humans: IgA, IgD, IgE, IgM and IgG. IgA and IgG are the most commonly used in therapeutics [25,27,28]. IgA are found in the mucosal membranes and help to prevent the colonization of mucosal pathogens. They are commonly found as dimers that take the shape of two Y’s bound together at the stem [27]. IgG are considered memory Abs and provide the main Ab-based immunity against pathogens and comprise approximately 80% of total pooled Abs within humans [28]. Some antibodies, such as IgD, are membrane-bound and involved in cellular signaling. Nearly all antibodies are glycosylated to assist in specificity and binding. There are two main types of Abs in the context of antigen binding. Monoclonal antibodies (mAbs) are identical in their sequence and specificity, while polyclonal antibodies (pAbs) are not identical in sequence [25]. MAbs are more often used as therapeutics and vaccines due to their higher specificity and homogeneity [29,30].

Antigen binding fragments (Fabs or Fvs) can often be used in place of whole antibodies. These fragments are one light chain and a section of a whole Ab that contains the variable domain. Another type of fragment called F(ab’)_2_, essentially two Fabs linked together, can also be isolated from Ab solutions (Figure 2B). Another Ab type that has become important in pharmaceutical development is small- or single-domain antibodies (sdAb) which were discovered in the family *Camelidae* (Figure 2C) [31,32]. These sdAb consist of the heavy chain homodimers that lack the light chains entirely. SdAbs are often cleaved at their disulfide bonds to separate the variable domain from the Fc region. The variable domain fragments are also called single variable domains (VHH, scFv) because their antigen-binding site is a singular variable domain of a heavy chain IgG. VHHs are also often called nanobodies due to their small size (Nanobody™ is a trademark of Ablynx N.V., Ghent, Belgium) [32].

**Figure 3:**
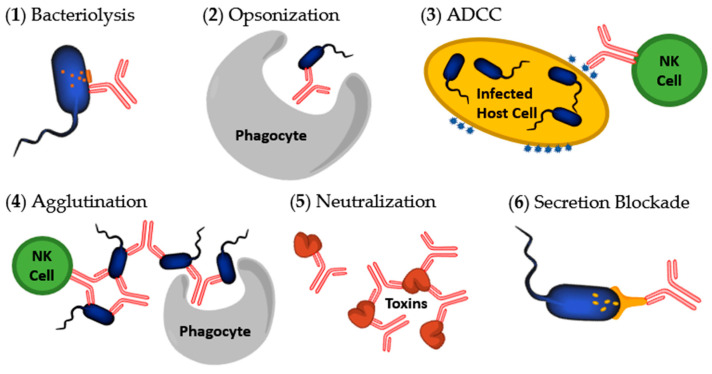
Mechanisms initiated by antibodies to destroy bacteria or toxins. (**1**) Bacteriolysis occurring after complement activation; (**2**) Opsonization by a macrophage or neutrophil after Fc sequence recognition; (**3**) Antibody-dependent cell-mediated cytotoxicity (ADCC) of an infected host cell; (**4**) Agglutination; (**5**) Neutralization of a bacterial secreted toxin; (**6**) Secretion blockade preventing T3SS proteins from being secreted. Image modified from [34].

There are multiple mechanisms by which an antibody can act to destroy or inactivate infectious agents. These include: (1) Complement-dependent bacteriolysis; (2) Opsonization or phagocytosis; (3) Antibody-dependent cell-mediated cytotoxicity (ADCC); (4) Agglutination; (5) Neutralization; and (6) Secretion blockade (Figure 3) [25,33,34]. Mechanisms 1–3 and 5 were the original biological effects that Abs were thought to perform. Agglutination (4) is not typically considered one of the mechanisms of Abs against bacterial pathogens because it leads to mechanism 2 or 3 but is included here for clarity. Secretion blockades (6) were only recently discovered in the context of T3SS inhibition research.

Complement activation occurs when Abs bind to an antigen of the bacteria or virus. This attracts the first component of the complement cascade and subsequently the classical complement system [33]. This activation results in pathogen death. The entire process is called complement-dependent cytotoxicity (CDC). CDC is divided into two distinct pathways for pathogen elimination; the Ab attracts and begins the formation of a membrane attack complex which then assists in bacteriolysis (1) or the Ab marks the bacteria for opsonization by neutrophils, macrophages, or other phagocytes (2) [33]. Opsonization is considered an indirect inactivation or inhibition of pathogenesis by Abs. This is because the Ab itself does not cause the halt of pathogenesis. Along with eventual bacterial death, complement activation also attracts inflammatory cells to the site [25].

ADCC is initiated by Abs that mark infected host cells for digestion or lysis (3). For example, after a pathogen invades a host cell, the host cell may break up some of the pathogen’s proteins and display them on the host membrane. Abs can bind to the displayed pathogen protein fragments and be recognized by natural killer (NK) cells. The NK cells then induce apoptosis of the infected host cell [35]. For clarity within this review, Abs that attach to antigens presented on the host cell will be considered marked for ADCC. When the bacterial cell is attached to the host cell then either ADCC or opsonization can be considered.

Agglutination occurs when the Ab binds to multiple foreign cells, clumping them together into large attractive targets for phagocytes (4). This eventually leads to opsonization (2). Agglutination also activates natural killer cells and initiates ADCC (3) [33,35]. Agglutination helps to prevent cell division in bacterial pathogens by physically lumping the cells together [25].

Neutralization is the process in which an Ab binds to an antigen, typically a toxin, causing physical or chemical inactivation of that antigen (5) [33]. Precipitation is another specific way in which antigens can be neutralized. Abs may bind to multiple soluble antigens to create larger, insoluble clumps that precipitate out of solution, once again making them attractive targets for phagocytes [33].

The most recently discovered mechanism for deactivating pathogens are secretion blockades. A secretion blockade occurs when the Ab binds to a secretion system and physically blocks the secretion of protein (6). This helps to prevent the bacteria from binding to host cells and infecting them [34]. This mechanism is initiated by Abs targeting the translocon or needle tip proteins of the T3SS. When Abs latch onto these proteins it can create a physical barrier, preventing the needle tip from attaching to the translocon correctly or the translocon from integrating into host cell membranes. One example of this phenomenon is seen by specific anti-LcrV Ab blocking the apoptotic action of LcrV, the *Yersinia* spp. needle tip, against human T-cells [36].

## 3. Antibodies as Pharmaceuticals

Edward Jenner, the father of the modern vaccine, used the blood serum of milkmaids who were immune to smallpox due to their exposure to cowpox to successfully vaccinate a child against smallpox [37]. This strategy was inspired by the way infants receive protection from maternal antibodies contained in their mother’s milk and was considered to be a passive immunization [23,38]. Since Jenner’s time, passive immunization has come to mean the direct administration of purified antibodies or antibody serum rather than human blood containing antibodies [30]. In contrast, active immunization is done with an antigen, such as a toxoid, or with whole-cell vaccines. The passive distinction comes from the lack of immune response required by the host to confer immunity.

Any vaccine can become ineffective over time due to mutations of the bacteria or virus, but when the protein’s sequence is highly conserved the chance of mutation is decreased [38]. Many components of the T3SS, such as the translocon, needle tip, needle subunits, and ATPases, are highly conserved between strains of a species of bacteria and often even between species of a particular genus [2]. This conservation allows for the immune response elicited by the injected antigen to have a high likelihood of recognition amongst different species or serovars of bacteria within the same genus. Vaccines targeting the T3SS also have shown promise as some subunit vaccines of the *Yersinia* needle tip protein have gone into clinical trials [39].

Nonspecific polyclonal human IgG pooled from 10,000 s of donors is known as intravenous immunoglobulin (IVIG) and has a wide variety of clinical uses that highlight the importance of using IgG as a therapeutic. IVIG contains antibodies with the ability to bind to a wide variety of antigens because it is pooled from so many donors [40,41]. IVIG is often used in immunocompromised patients as a prophylactic to prevent infection [42,43], or in those struggling with active infections [44,45]. If a grouping of non-specific antibodies can help to prevent and treat disease, then antibodies designed specifically to act upon pathogens should be able to do the same at a higher specificity without some of the common side effects and complications of IVIG, such as fever, migraines, anxiety, nausea, and vomiting [46,47]. Other benefits to designed antibody therapeutics are the consistency in their preparation, homogeneity of contents, and ease of engineering [48].

Antibodies have recently come into prominence as therapeutics. To date, there have been approximately 85 mAbs approved by the FDA for use as immunotherapy and 80 that have been approved by countries within the European Union [49]. Abs used as therapeutics are often administered via intravenous (IV) injections, intramuscularly, or parenterally [28,47]. The route of administration can be very important for the effectiveness of therapeutic Abs. For example, Sécher et al. showed the administration of anti-PcrV pAbs is more effective at treating *Pseudomonas aeruginosa* infections when done via airways than through parenteral injections [50]. This is likely due to the localization of the therapeutic at the area of infection as *P. aeruginosa* infects lung epithelial tissue. In the case of a gastrointestinal pathogen, such as *E. coli* or *Shigella* spp., an oral route of administration may be more effective than IV injections [51]. Hill et al. showed that local administrations of anti-LcrV and F1 Abs could be used as a *Yersinia* infection treatment or prophylactic, while injections of the same Abs could only act preventatively when administered multiple weeks in advance [52]. Their research suggests that this method of localization to the lung could be effective as a fast-acting post-exposure treatment for pneumonic plague.

Breastfeeding has been shown to reduce the risk of infant diarrheal disease, of which *E. coli* is a main culprit, from 76% to 26% [53]. Loureiro et al. showed that passive immunization of infants with anti-T3SS Abs via breastfeeding protects them against infection with two strains of EPEC. Abs targeting three separate T3SS-related proteins were discovered in infants in areas where EPEC-caused diarrhea is endemic. These Abs were isolated from blood samples and shown to decrease host cell binding of EPEC. They also acted as potent opsonins for killing EPEC [18]. Both of these are evidence for the protective properties of anti-T3SS Abs against Gram-negative pathogenic bacteria.

No studies on Abs protecting against a bacterial challenge have been performed in human infants. There have, however, been studies in baboons. Kapil et al.’s study of maternal vaccination against *Bordetella pertussis*, the causative agent of whooping cough, showed that Abs transferred via breastfeeding were sufficient to protect against a *B. pertussis* infection. Infant baboons born to vaccinated mothers did become highly colonized with the pathogen but did not exhibit signs of disease and cleared the infectious bacteria approximately three weeks after bacterial challenge. The infants born to non-vaccinated mothers, on the other hand, exhibited severe disease symptoms and all but one were euthanized due to the severity of symptoms [54].

### 3.1. Challenges of Anti-T3SS Antibody Therapies

Compared to small molecules, Abs have much higher specificity and affinity to their targets and can easily be identified as drug candidates. They also have longer half-lives due to lower CYP450 metabolism and high serum stability [29]. Along with these benefits, Abs are twice as likely to be approved and moved to market once entering in-human trials than small molecules [55]. All of these traits make Abs desirable candidates for drug development. Unfortunately, there are some downsides to Ab therapeutics when compared to small molecules. Abs typically cannot penetrate cell membranes, which means that intracellular targets are unavailable to them [56]. This issue has inspired techniques to engineer cell-penetrating Abs and antibody fragments, but these increase the cost of production [57]. Abs also have a higher cost of production than small molecules [58].

When discussing antibody therapies the risk of host rejection and severe side effects must also be considered. A higher dosage leads to a higher risk of adverse side effects or effects on plasma viscosity. This is most commonly an issue with IVIG where replacement treatments are approximately 200–400 mg/kg given every two to three weeks continuously and acute treatments can reach 2000 mg/kg monthly. The large doses in IVIG treatment are less desirable when compared to the treatment of acute infections with mAbs that range closer to 5–50 mg/kg [59]. This can be contrasted, however, with the risk of anti-antibody formation.

Prolonged use of biologics, particularly mAbs, can cause the development of anti-drug antibodies (ADAs). IVIG is at low risk for ADA neutralization because the solution of Abs is from separate donors and less likely to contain high concentrations of any particular antibody. In comparison, mAbs are a singular Ab, meaning only one type of ADA is required for neutralization. Once ADA are present in the patient the treatment is often no longer administered due to changes in pharmacokinetics (PK) and the risk of allergic reactions. ADAs can function by a variety of different mechanisms. Some ADAs, called binding ADAs, increase clearance by complex formation while others, neutralizing ADAs, increase clearance and neutralize by binding to the epitope associated with the therapy [59]. Once a biologic therapy reaches preclinical status, the effect of ADAs on immunogenicity and PK must be considered and determined. The risk of an allergic reaction due to ADA formation must also be documented. The production of ADAs against an anti-T3SS antibody would not necessarily be prohibitory to its success. The risk of ADAs against mAbs currently on the market range from 0% to 89%, although the majority are under 10% [59].

Clinical trials of antibodies used to treat Gram-negative infections, including sepsis, have had limited success in the past. IVIG has been approved to treat sepsis, but there is a high degree of heterogeneity in the results of treatment, leading to unclear guidelines for dosing and preparation [48]. MAbs have faced even larger difficulties. A clinical trial on the effects of anti-lipopolysaccharide (LPS) IgG revealed no therapeutic benefit in any clinical parameter measured from either the administration of anti-LPS mAbs or endogenously produced antibodies [60]. It is unclear whether therapeutic antibodies targeting the T3SS will encounter the same difficulties. A clinical trial on an anti-PcrV F(ab’)_2_ was tested for its efficacy at preventing and treating sepsis due to *P. aeruginosa*. The treatment did significantly prevent onset of sepsis, but protection was not considered complete. When administered after the onset of sepsis there was a slowing of disease progression, resulting in a decreased rate of septic shock. Presence of the pathogen, however, was not significantly decreased by the administration of anti-PcrV F(ab’)_2_ [61]. This suggests that anti-T3SS antibody treatments alone may not be enough to treat or prevent disease.

The research presented in this review is focused on the use of anti-T3SS Abs as individual therapies, but combination therapies are more likely in practice [62,63]. Combination therapy may reduce drug resistance emergence by allowing for reduced dosages and treatment duration of antibiotics [64]. Evidence of this synergistic approach can be seen in Secher et al.’s study of an anti-*P. aeruginosa* mAb with meropenem, a broad-spectrum antibiotic. They found treatment with this combination led to an additive effect. When the combination was given to patients with meropenem-resistant infections the mAb efficacy was comparable to treatments with the mAb alone against meropenem-sensitive infections [65]. Le at al. demonstrated that MEDI3902, an anti-T3SS mAb, showed enhanced activity when treating *P. aeruginosa* infections when administered in combination with a subtherapeutic dose of meropenem [66]. These studies, along with others discussed in this review, are evidence of practical applications of anti-T3SS Ab therapeutics when used in combination with traditional antibiotic approaches.

### 3.2. Strategies to Enhance Antibody Production

The costs associated with antibody production and isolation can be a limiting factor in their use. Strategies to enhance and reduce the cost of antibody therapeutics is an ongoing research area. Since the mid-1970s, hybridomas have been the main technology used in mAb production. The most common cells used for recombinant mAb production are Chinese hamster ovary (CHO) cells [67,68]. Identification and engineering of high-Ab production cell lines have long been a challenge. CHO cells are known to produce antibodies at approximately 1 g/L after optimization [68,69]. Factors that play a role in the success and efficiency of cell lines include the time until desired cell density is reached, the duration of production time allowing for antibody harvesting, and the overall titer of antibody produced [67]. Itoh et al. found that suppression of apoptosis-associated genes allows for longer culture times and therefore higher Ab titers [70]. The overproduction of proteins involved in protein folding, such as CHOP, have also shown improvement in the viability of antibodies produced [71]. Sittner et al. developed fluorescence-activated cell sorting (FACS) to produce Abs more effectively against LcrV, a T3SS needle tip protein. This technique is intended to be used in conjunction with hybridoma mAb production. FACS increased yields of mAbs by 773%, from 22 to 170 positive clones per spleen [72].

MAbs are routinely produced by hybridomas; replacing them with bacteria is a strategy to reduce the cost of production [73,74]. MAbs produced in bacteria face challenges of protein aggregation, inefficient folding, and low yields [75]. Zhou et al. produced a full-length mAb in *E. coli* by fusing the signal peptide of disulfide oxidoreductase to the N-terminus of the heavy chain of the mAb to assist in secretion into and accumulation in the periplasm. This tag helped to reduce the bottleneck in production caused by inefficient heavy chain secretion [73]. Plants have also been used to produce Abs cost-effectively. Saberianfar et al. isolated sdAbs that target a T3SS effector from tobacco (*Nicotiana benthamiana*) leaves at a level of 1% to 3% of total soluble protein [74].

## 4. T3SS Components Targeted by Antibodies

The two most common targets of antibodies against the T3SS are the needle tip and translocon proteins. The binding sites of Abs to either of these proteins are easily accessible to the antibodies due to their extracellular location. Binding marks the bacteria for opsonization or creates a secretion blockade to prevent effectors from entering the host cell. Part of the basal body is available extracellularly and has been used as an Ab target. Secreted effector proteins are also common targets of Ab therapies because they are often toxins and humans naturally produce Abs against them. There has been limited experimentation targeting regulatory proteins to prevent expression of the T3SS.

### 4.1. Needle Tip

**Figure 4:**
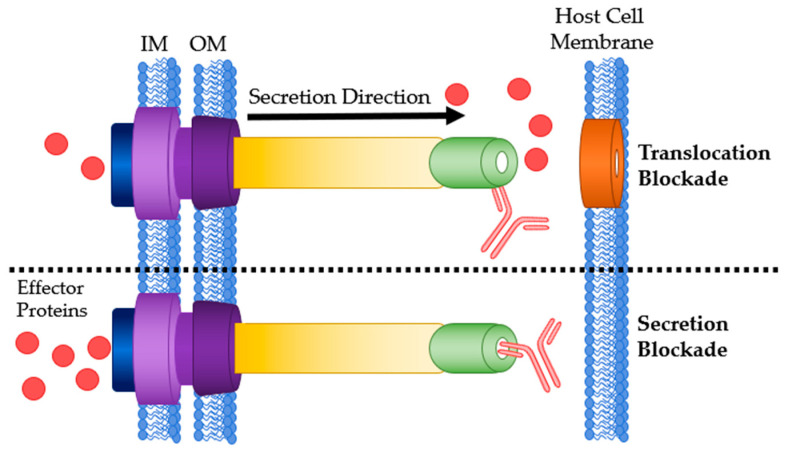
Antibody binding sites on the T3SS needle tip. **Top**: Ab bound to the needle tip protein resulting in a translocation blockade. **Bottom**: Ab bound to the needle tip protein physically blocking any effector secretion. IM: bacterial inner membrane; OM: bacterial outer membrane.

The needle tip protein of many T3SSs, also called the V antigen, causes mammalian hosts to produce specific IgG. Kinoshita et al. found relatively high and comparable antibody titers in human sera against V-antigen homologs from five different bacterial species: *P. aeruginosa*, *Y. pestis*, *Photorhabdus luminescens*, *Aeromonas salmonicida* and *Vibrio parahaemolyticus* [76]. Abs targeting the T3SS needle tip will likely adopt the secretion blockade mechanism of pathogenesis prevention [34]. This has two variations: translocation blockade or a true secretion blockade (Figure 4). Translocation is defined as secretion directly into a host cell while secretion is an expulsion of protein through the T3SS needle. When an anti-needle tip Ab binds to the needle tip it can create a physical barrier between the tip and the translocon. This barrier prevents secreted effectors from directly entering the host cell and instead are secreted into the extracellular matrix. True secretion blockades occur when the Ab binding prevents the effector proteins from exiting the needle. When designing anti-needle tip Abs, a true blockade style inhibition is desirable because the effector proteins are never released.

Vaccines for bubonic plague have existed since the late 19th century [77,78]. In 1958, researchers noticed V antigen was present in pathogenic strains of *Yersinia*, but not in non-pathogenic strains [79]. Passive transfer of anti-V antigen antisera limits infection by *Yersinia pestis*, the bacterial agent of the plague [80]. Motin et al. confirmed this immunogenicity by testing mAbs and recombinant Abs against V antigen in 1994 [81]. Once the T3SS was discovered, the V antigen was determined to be LcrV, the needle tip protein of the *Y. pestis* T3SS [82,83]. Abs targeting LcrV were sufficient to prevent translocation of effector proteins by the *Y. pestis* T3SS [84]. Since this time, there has been an explosion of research regarding anti-LcrV antibodies [72,85,86,87,88,89,90].

Miller et al. demonstrated the importance of cross-strain and cross-species compatibility when designing therapeutic Abs. PAbs and mAbs against one strain of *Y. pestis* LcrV were able to bind the LcrV of two other strains of *Y. enterocolitica*, but no other species or strains tested [85]. Ivanov et al. confirmed that anti-LcrV mAbs were sufficient to directly prevent the secretion of Yop effector proteins. Comparison of IgG mAbs to deglycosylated F(ab’)_2_ and unmodified Fab revealed that the mAb did not require opsonophagocytosis to neutralize Yop translocation [86]. Their work provided a foundation to show that anti-needle tip Abs were acting as a secretion blockade and not a translocation blockade [34].

*P. aeruginosa* has a needle tip protein that is a homolog to LcrV called PcrV [2]. There is a wide breadth of knowledge on anti-PcrV Abs [61,66,91,92,93,94,95,96,97,98,99,100,101]. The use of anti-PcrV Abs as a vaccine or therapeutic is a topic of interest for many researchers. Taking inspiration from the successes of anti-LcrV therapeutics, Shime et al. investigated anti-PcrV polyclonal IgG and F(ab’)_2_ [61]. Anti-PcrV whole IgG significantly improved the survival rate of mice infected with otherwise lethal doses of *P. aeruginosa* and protected against septic shock in an airspace-infected rabbit model. F(ab’)_2_ derived from the IgG was also tested and the results were comparable.

Polyclonal anti-PcrV IgG as a passive immunization has been evaluated in other models. Burned mouse model results showed that anti-PcrV IgG was significantly better than control IgG at increasing survival rates of mice challenged with a lethal dose of *P. aeruginosa* [91]. Anti-PcrV Abs showed increased effectivity in combination therapy with three separate antibiotics against acute *P. aeruginosa* infection. The combination showed better effectivity than any of the antibiotics or Ab when administered alone [92]. IgY is chicken egg yolk immunoglobulin that is functionally homologous to mammalian IgG. IgY does not react with the mammalian complement system. This reduces the inflammatory response during administration, which makes it an attractive alternative to mammalian IgG [102]. Ranjbar et al. have recently shown that anti-PcrV IgY was more protective against *P. aeruginosa* acute pneumonia and burn-associated infections than control IgY. IgY was comparable to IgG and could serve to be a more affordable alternative in the future [93].

In 2002, Frank et al. tested anti-PcrV mAbs from 80 strains of *P. aeruginosa* to determine which would confer the highest immunoprotection. T3SS secretion assays were performed to determine which mAbs could prevent the translocation of ExoU, a T3SS effector of *P. aeruginosa*. The mAbs showing T3SS inhibition were evaluated for their ability to protect against an otherwise lethal challenge of *P. aeruginosa* in a mouse survival assay. MAb166 was the only antibody tested that showed dose-dependent T3SS inhibition and immunoprotection [94]. De Tavernier et al. has turned to computational methods to find more effective anti-PcrV nanobodies. Three hundred and sixty-one bivalent and biparatopic nanobodies were screened computationally for their ability to cause secretion blockades. T3SS secretion inhibition assays using these nanobodies was performed, followed by mouse survival assays. The most potent nanobody, 13F07-5H01, was effective as a prophylactic up to 24 h after administration, the longest time point tested [95].

Some anti-PcrV mAb therapies have gone into human trials. One of these, termed KB001-A, is a human PEGylated IgG monoclonal anti-PcrV Fab [96,97,98]. In France, KB001-A underwent phase I and II clinical trials for ventilator-associated *P. aeruginosa* and was considered to be safe and well-tolerated. It did not advance to phase III trials due to a lack of evidence that it reduced pulmonary disease exacerbation in mechanically ventilated patients [103,104]. KB001-A also underwent phase II clinical trials in the US for treatment of chronic pneumonia in cystic fibrosis patients and performed well in safety-based phase I trials but did not continue to phase III [105].

More recently, an alternative anti-PcrV mAb, MEDI3902, has entered human clinical trials. This mAb is bispecific, targeting both PcrV and Psl exopolysaccharide, an anti-biofilm formation target. MEDI3902 showed a dose-dependent survival increase and a decrease in bacterial load in both rabbit and mouse *P. aeruginosa* challenge models. MEDI3902 also reduced lung inflammation caused by bacterial colonization [99]. Le et al. showed MEDI3902 was effective as a treatment and a prophylactic for acute blood and acute lung *P. aeruginosa* infections. Combination therapy with a subtherapeutic dose of the antibiotic meropenem enhanced effectivity [66]. MEDI3902 performed well in phase I clinical trials and is currently undergoing phase IIb trials in the US [106,107].

EspA is the needle tip in the T3SS of *E. coli* [2]. In 2006, recombinant anti-EspA pAbs were shown to reduce actin cytoskeleton rearrangement of the host cell but did not show any reduction of bacterial adhesion [108]. This was the first report of anti-EspA Abs showing inhibitory effects upon the T3SS. Girard et al. investigated the effectivity of bacterial adherence inhibition with IgY to multiple *E. coli* T3SS-related colonization factors, one of which was EspA. Unfortunately, the anti-EspA polyclonal IgY did not significantly reduce bacterial adhesions in multiple strains of pathogenic *E. coli* [109]. The Girard results were disputed when Cook et al. published that anti-EspA IgY and rat IgG reduced adherence of *E. coli* to HeLa cells and prevented T3SS secretion [110].

Yu et al. discovered a novel anti-EspA mAb, 1H10 that provided protection for mice in a survival assay and blocked actin polymerization within host cells [111]. In 2014 Praekelt et al. researched the five major variants of EspA to create over 200 mAbs. Three separate mAbs reacted with multiple EspA variants [112]. While this research was intended to create a better *E. coli* diagnostic test, the results could be applied to treat or prevent *E. coli* infections.

*Salmonella enterica* serovar Enteritidis (*S*. Enteritidis) causes a large portion of food-related illnesses around the world. *Salmonella* has two T3SSs. The first, called T3SS1, is encoded by the SPI-1 pathogenicity island and is used for host cell entry. The second, called T3SS2, is encoded by the SPI-2 pathogenicity island and is used for further pathogenesis once inside the host cell. SipD is the T3SS1 needle tip protein [2]. Desin et al. have shown that anti-SipD pAbs in sera protected human Caco-2 cells from the entry of *S*. Enteritidis [113].

The needle tip protein in *Shigella* spp. is IpaD [2]. Barta et al. showed that small molecule binding to IpaD induced conformation changes. These changes are accompanied by a significant reduction in the invasive potential of *Shigella* [114]. A panel of anti-SipD nanobodies was tested for their binding affinity and their epitopes were determined. Nanobodies targeting the same section of the needle tip protein resulted in the same conformational change [115]. This research supports the theory that anti-needle tip antibodies may be able to create secretion blockades without physically blocking the effectors from being secreted.

### 4.2. Translocon

**Figure 5:**
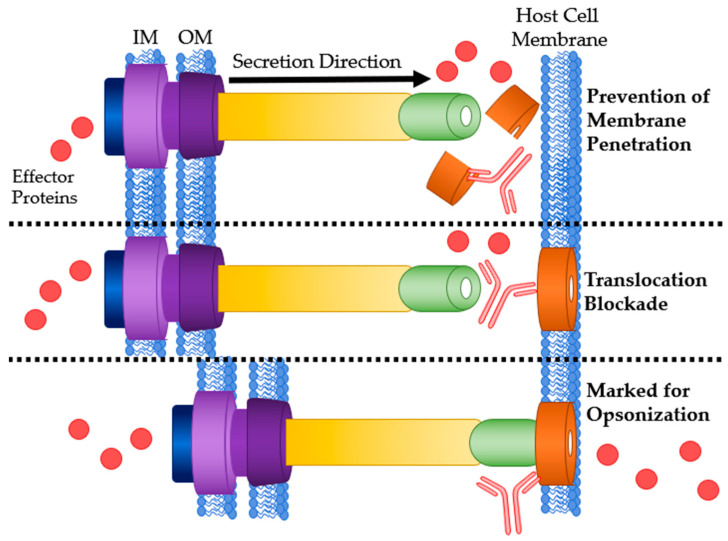
Mechanisms of Ab interaction with T3SS translocon proteins. **Top**: Ab binding to translocon proteins after secretion preventing host membrane penetration and pore formation. **Middle**: Physical blockage of translocation by Ab binding. **Bottom**: Ab bound to the translocon marking it for opsonization or ADCC. IM: bacterial inner membrane; OM: bacterial outer membrane.

The translocon is made of two proteins that are secreted and subsequently enter the host cell membrane and form a pore. They then link to the needle tip to complete the channel between the pathogen and host cells. Humans naturally produce antibodies against translocon proteins [22]. Abs that target these proteins adopt similar mechanisms to anti-needle tip Abs. They can bind at three time points: before integration into the host membrane, preventing pore formation; after pore formation but before the needle tip has attached, therefore blocking it from attaching and creating a translocation blockade; or once the T3SS is active, mark the cell for ADCC or opsonization (Figure 5).

The *Y. pestis* T3SS translocon is composed of the proteins YopB & YopD [2]. Ivanov et al. investigated these proteins and their potential for therapeutic use in the treatment and prevention of *Y. pestis* infection. Active and passive immunization protected against otherwise lethal injections of *Y. pestis* in mice [116]. They posited YopD was the dominant immunogen due to the higher titers of Abs and that passive immunization with anti-YopD Abs alone would be enough to confer protection.

The translocon of *E. coli*’s T3SS is formed with two proteins, EspB and EspD [2]. A study of Brazilian children showed that those with naturally occurring anti-EspB Abs were less likely to have a severe EHEC infection [117]. Maternal vaccination with EspB affords passive immunization of offspring with anti-EspB Abs. These Abs are effective at reducing risk for *E. coli* infection and increasing infection survival rates [24]. Similar experiments using mice have shown comparable survival results. Placebo-vaccinated mothers had offspring with significantly higher plasma urea concentrations, a marker of renal failure [118].

*Salmonella* Enteritidis’ translocon is comprised of proteins SipB and SipC [2]. Although there is limited information about antibodies targeting these proteins there is evidence of a mAb indirectly preventing the formation of the T3SS-1 translocon in *Salmonella*. The mAb Sal4 targets a surface polysaccharide of *Salmonella* named O antigen. Forbes et al. observed that Sal4 appeared to interfere with flagellum-based motility and T3SS-mediated entry of the host intestinal epithelium [119]. Interference with host cell entry by Sal4 was due to inhibition of the T3SS. Direct interaction of SipB or SipC with Sal4 was not tested, but other T3SS components and effectors were eliminated as antigens for Sal4 [120].

### 4.3. Basal Body

**Figure 6:**
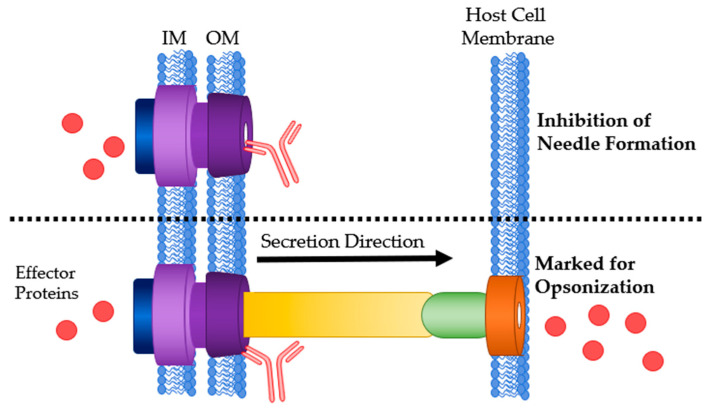
Mechanisms of Ab interaction with the T3SS basal body. **Top**: Ab bound to the outer membrane ring of the T3SS basal body preventing needle formation. **Bottom**: Ab bound to the basal body marking it for opsonization or ADCC. IM: bacterial inner membrane; OM: bacterial outer membrane.

The basal body of the T3SS contains four major components. These include an ATPase that powers secretion, the lower ring within the inner bacterial membrane, an export apparatus that is visible between the bacterial membranes, and the upper ring located in the outer membrane of the bacterial cell. Of these components, only the upper ring of the basal body is exposed to the extracellular matrix. Research regarding therapeutic anti-upper ring Abs has focused on Abs that will mark the T3SS, and therefore the pathogen, for opsonization [121]. There is a possibility that the needle formation could be inhibited if Abs bind to the correct area of the upper ring (Figure 6). YscC makes up the upper ring of the *Y. pestis* T3SS basal body [2]. Goodin et al. have shown that passive immunization with anti-YscC pAbs induced the mouse immune system to produce more anti-YscC Abs but did not provide sufficient protection against a lethal *Y. pestis* challenge in comparison to an F1 & LcrV protein-based vaccine [121]. The outer membrane ring of the T3SS basal body shares high similarity to other outer membrane proteins in secretion systems unrelated to pathogenesis [122]. The similarity could, in theory, allow for Ab binding to secretion systems on commensal bacteria. This potentially reduced specificity along with the lack of protection in the Goodin study highlights the challenge of using the basal body as a target for therapeutic Abs.

### 4.4. Effector Proteins

The critical function of the T3SS is to secrete proteins directly into host cells. These effector proteins have a wide variety of mechanisms and purposes [6,7,8,9]. Knockout or mutations of some effectors can result in attenuation of virulence and reduced pathogenesis, making them attractive targets [123]. The majority of effector proteins are translocated into the host cell and are unable to leave. This creates a challenge in using antibodies against these particular effectors [56]. Some effectors, however, can transverse and exit the host cell after translocation, making them available for neutralization by antibodies. Other effectors may be presented on the surface of host cells. These proteins are more accessible for targeting by antibodies.

#### 4.4.1. Antibodies Targeting Extracellularly Available Effectors

**Figure 7:**
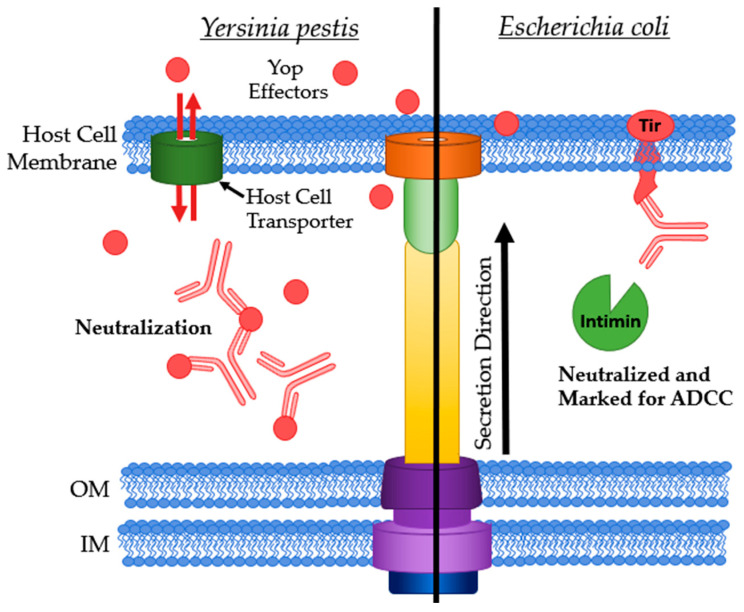
Antibodies targeting extracellularly available effector proteins. **Left**: Yop effectors being neutralized when in the extracellular matrix. **Right**: Ab bound to Tir marking the host cell for ADCC and neutralizing Tir by preventing intimin binding. IM: bacterial inner membrane; OM: bacterial outer membrane.

*Yersinia* outer proteins (Yops) are a class of T3SS effectors in *Y. pestis*, *Y. enterocolitica* and *Y. pseudotuberculosis*. Some Yops are present both internally and in the extracellular space (Figure 7, Left). Akopyan et al. observed the presence of Yops outside host cells and found that YopE is localized to the surface of host cells, but not necessarily where the T3SS is attached. They hypothesized that some Yops (e.g., YopE) can enter host cells in a T3SS-independent manner by hijacking host transporters while others must utilize the T3SS (e.g., YopH) [124].

Ivanov et al. discovered that anti-YopE Abs inhibited bacterial infection but to a lesser extent than Abs against the translocon proteins, YopB, and YopD [116]. Later, Singh et al. vaccinated mice against rVE, a YopE-LcrV fusion protein, using both active and passive approaches. Active immunization with the protein conferred high titers of Abs against both antigens. The serum from vaccinated mice was used to vaccinate another batch of mice. These passively immunized mice showed nearly 90% survival against a lethal challenge of *Y. enterocolitica* with the majority showing no signs or symptoms of infection even after necropsy [125]. YopE specific CD8^+^ T cells are naturally occurring immune cells coated in Abs that recognize YopE. Immunization of mice with these cells was 60% protective against mucosal and systemic *Y. pseudotuberculosis* infection in a survival assay. YopE specific CD8^+^ T cells, not just anti-YopE Abs, are required for protection against infection [126].

The essential effector YopM is a modulator of kinases PRK1 and PRK2. This modulation eventually leads to a reduction in pro-inflammatory cytokines lessening the effectivity of the host immune response [8]. YopM is the first effector to be recognized as a bacterial cell-penetrating protein as it can leave and re-enter host cells after translocation [127]. Neutralization of YopM would in theory restore the host cytokine response as well as precipitation of YopM, resulting in the recruitment of phagocytes to the infection site (Figure 7, Left). Rüter et al. have isolated an anti-YopM mAb that binds to multiple strains of pathogenic *Yersinia*’s YopM but not to YopM from non-pathogenic strains [128]. This specificity may be beneficial when designing therapeutics and diagnostics.

The translocated intimin receptor (Tir) is one of the first effector proteins translocated by *E. coli*’s T3SS into host cells. After folding, Tir integrates into the host membrane to provide a pedestal for adhesion via intimin binding [129]. This extracellular expression allows for Abs to bind without having to cross the eukaryotic membrane (Figure 7, Right). Girard et al. found that anti-Tir IgY were effective at preventing bacterial adhesion to host cells in a porcine model against both porcine and human strains of enteropathogenic *E. coli* (EPEC) [109]. Ruano-Gallego et al. assessed the potential of an anti-Tir nanobody, TD4, as a potential treatment or prophylactic for EHEC infections. TD4 inhibits the attachment of EHEC to HeLa cells and reduces adherence to human colonic mucosa [130]. Anti-intimin IgY was effective at reducing adherence of both EPEC strains to host gut epithelial tissue in an ileal loop assay as well as in oral administration of the IgY [109]. Kühne et al. purified nanobodies binding to the Tir-binding domain of intimin [131]. Saberianfar et al. investigated the Tir-binding domain of intimin as a target to isolate sdAbs from tobacco leaves. These sdAbs were used to design a chimeric Ab, V_H_H10-IgA. This Ab inhibited four strains of EHEC from adhering to host cells, with three of the four completely inhibited [74]. V_H_H10-IgA’s cross-serotype inhibition of bacterial adhesion is highly promising for future studies.

#### 4.4.2. Adjuvating Antibodies Targeting Effectors

Effector proteins that are only present within the host cell become available upon cell lysis [25]. Abs targeting these effectors will not be able to prevent T3SS formation or secretion but will add to the host immune response [132,133]. Some infected host cells will also participate in antigen presentation. This is the process of breaking up non-native proteins, such as T3SS effectors, and displaying the fragments on the host membrane surface so that Abs can access them [25]. Although not as immunoprotective as T3SS inhibitory Abs, adjuvating Abs can be important to increase natural host immune response. Their use is common in combination with inhibitory Abs or as diagnostics.

Desin et al. were inspired by traditional research on T3SS protein-based vaccines in cattle and other ruminants, the main animal reservoir for Shiga toxin-producing *E. coli* (STEC) [18]. Passive immunization using uncharacterized rabbit-produced sera was sufficient to block adherence of *E. coli* to host cells [134]. Desin et al. also tested antisera containing pAbs against three effectors: Tir, EspF, and NleA (EspI), along with the needle tip protein, EspA, and a translocon component, EpB. EspF assists in host cytoskeleton rearrangement and inhibits host cell apoptosis. NleA localizes to the Golgi apparatus in the host cell and disturbs ER to Golgi transport. Desin et al. showed Abs against either EspF or NleA inhibited bacterial adherence of two STEC strains (STEC_O103_ & STEC_O157_).

ExoS, exoenzyme S, is an effector protein secreted by the *P. aeruginosa* T3SS. ExoS, along with three other effectors: ExoU, ExoY, and ExoT, assist in the prevention of wound repair in the host by reducing the immune response and causing damage to host mucosal membranes [135]. Knockout and mutations of ExoS result in reduced pathogenesis of *P. aeruginosa* [123]. Corech et al. examined the serum of patients with *P. aeruginosa* infections and found they universally had significant titers of IgG against PopB, PcrV, and ExoS [136]. These antibodies may be viable candidates for pharmaceutical development.

IncA is an effector secreted by the *C. trachomatis* T3SS and generates robust IgG responses in humans. *C. trachomatis* enters host cells where it replicates within vacuoles called inclusions. IncA has a role in the homotypic fusion of these inclusions. Pathogenic *C. trachomatis* has attenuated host cell invasion in the presence of anti-IncA Abs [137]. Tsai et al. sequenced IncA from multiple *C. trachomatis* isolates and found that they were nearly identical to all human serotypes sequenced thus far, suggesting that anti-IncA Abs should react with IncA from multiple serotypes. Anti-IncA Abs were found in 52% of urine samples and 71% of genital samples from *C. trachomatis*-infected patients [138]. Although further research is needed to confirm the immunogenicity of IncA, these results support the use of anti-IncA Abs as diagnostic or therapeutic antibodies.

An effector secreted by the *Salmonella* T3SS2 is SpiC, also called SsaB. SpiC interferes with host cell trafficking and knockouts show attenuated virulence and decreased T3SS2 activity [139]. Geng et al. developed seven anti-SpiC mAbs. These mAbs bound specifically to SpiC and not to the His or GST, both of which were used in the mAb isolation process [140]. Immunogenicity data for these mAbs was not presented, but there is potential for their use as therapeutics or diagnostic tools.

#### 4.4.3. Antibodies Targeting Intracellular Effectors and Transcription Factors

**Figure 8:**
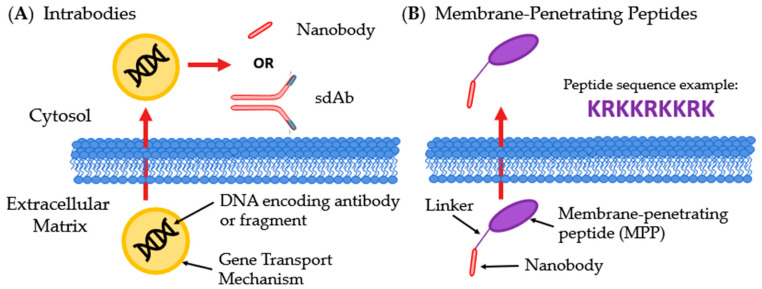
Innovative methods for intracellular delivery of Abs. (**A**) Intrabodies enter the host cell containing DNA encoding a therapeutic Ab or antibody fragment, most often a sdAb or nanobody; (**B**) Membrane-penetrating peptides are linked to Abs or fragments, commonly nanobodies, and allows for transversing membranes.

Delivery of antibodies into cells is required to access intracellular targets. One method to overcome issues of cell penetration is to express the antibody within the cell. Intrabodies are internally expressed antibodies. Gene transport mechanisms are used to deliver the DNA encoding the therapeutic antibody or antibody fragment inside the cell where it can be transcribed (Figure 8A) [31,32,141]. Another method of internalizing Abs is to pair them with a membrane-penetrating peptide (MPP). Attaching an MPP to an antibody or antibody fragment allows for the therapeutic antibody or fragment to physically transverse the membrane. The MPP destabilizes bacterial cell membranes and enables the fused protein to traverse the outer membrane (Figure 8B) [142].

SpvB is a cytotoxic effector secreted through the *S.* Typhimurium T3SS2 into the host cell from the vacuole containing the pathogen. Once in the host, it catalyzes ADP-ribosylation of actin and eventually causes host cell apoptosis. Alzogaray et al. have developed a nanobody that binds with a high affinity to SpvB. The nanobodies were expressed as intrabodies to neutralize the effector within the host cell [143]. The anti-SpvB antibodies stopped the action of SpvB in an ATP-induced actin polymerization fluorescence-based assay in vitro and in RAW macrophages [143].

Targeting a transcriptional regulator of the T3SS could prevent the proteins that make up the T3SS from being produced in the first place [144]. This method of pathogenesis prevention is not common, as the antibody in question would have to enter the pathogen rather than a eukaryotic host cell. SpuE is a transcriptional regulator of the T3SS in *P. aeruginosa* and regulates the expression of ExsA, a master regulator of the T3SS [2], via inhibition of the exsCEBA promoter. Zhang et al. derived an anti-SpuE nanobody fused with a membrane-penetrating peptide (scFv5-MPP) to assist in delivering the nanobody into the bacterial cell [145]. scFv5-MPP allosterically inhibits the expression of the T3SS and attenuates virulence of *P. aeruginosa* in a *C. elegans* animal gut infection model. X-ray crystallography and molecular dynamics simulations were used to observe conformational changes upon antibody binding to SpuE. This conformational change may cause a reduction in spermidine uptake by *P. aeruginosa* leading to attenuation of virulence similar to physical neutralization [145]. A similar antibody, Mab 4E4, protects A549 cells against *P. aeruginosa* infection by reducing T3SS expression and polyamine uptake. Wang et al. have recently tested Mab 4E4 in vivo and found that single injection vaccination with the antibody significantly increase survival rates of mice given a four-fold lethal dose of *P. aeruginosa* infection and protected them from severe alveolar destruction [146]. These examples validate the strategy of anti-transcriptional regulator antibodies as therapeutics or prophylactics against T3SS-utilizing bacteria.

## 5. Conclusions

A diverse array of antibodies has been used to inhibit the T3SS. These Abs bind to proteins of the injectisome including the needle tip, translocon, basal body, and effectors. Transcriptional regulators of the T3SS have also been targeted to prevent the formation of the T3SS, but as they are intracellular targets and require innovative cell-penetrating Abs. These can include entrapping DNA encoding the Ab in intrabodies or attachment of membrane-penetrating proteins to the Ab. Once the Ab has reached its target there are multiple mechanisms it can employ to attenuate virulence or increase the host immune response. In general, these Abs neutralize the effectors, mark the bacterial cell for phagocytes to attack, or mark the infected host cell for ADCC by NK cells. Sometimes Abs adopt more specific mechanisms. For example, when targeting the needle tip or translocon the Ab can physically block the secretion of effectors in a secretion blockade. Several anti-T3SS mAbs have advanced to clinical trials, but none have yet made it to market. As we learn more about how these antibodies function there will undoubtedly be potential for improvement of their therapeutic effects, cost of production, and the ease of their delivery.

## References

[1] Lara-Tejero M., Galán J.E. (2019). The injectisome, a complex nanomachine for protein injection into mammalian cells. EcoSal Plus.

[2] Hu Y., Huang H., Cheng X., Shu X., White A.P., Stavrinides J., Köster W., Zhu G., Zhao Z., Wang Y. (2017). A global survey of bacterial type III secretion systems and their effectors. Environ. Microbiol..

[3] Pendergrass H.A., May A.E. (2019). Natural product type III secretion system inhibitors. Antibiotics.

[4] Cornelis G.R., Van Gijsegem F. (2000). Assembly and function of type III secretory systems. Annu. Rev. Microbiol..

[5] Cheung M., Shen D.K., Makino F., Kato T., Roehrich A.D., Martinez-Argudo I., Walker M.L., Murillo I., Liu X., Pain M. (2015). Three-dimensional electron microscopy reconstruction and cysteine-mediated crosslinking provide a model of the type III secretion system needle tip complex. Mol. Microbiol..

[6] Hume P.J., Singh V., Davidson A.C., Koronakis V. (2017). Swiss army pathogen: The *Salmonella* entry toolkit. Front. Cell Infect. Microbiol..

[7] Mattock E., Blocker A.J. (2017). How do the virulence factors of *Shigella* work together to cause disease?. Front. Cell Infect. Microbiol..

[8] Zhang L., Mei M., Yu C., Shen W., Ma L., He J., Yi L. (2016). The functions of effector proteins in *Yersinia* virulence. Pol. J. Microbiol..

[9] Morrow K.A., Ochoa C.D., Balczon R., Zhou C., Cauthen L., Alexeyev M., Schmalzer K.M., Frank D.W., Stevens T. (2016). *Pseudomonas aeruginosa* exoenzymes U and Y induce a transmissible endothelial proteinopathy. Am. J. Physiol. Lung Cell Mol. Physiol..

[10] Marshall N.C., Brett Finlay B. (2014). Targeting the type III secretion system to treat bacterial infections. Expert Opin. Ther. Targets.

[11] Matsuda S., Okada R., Tandhavanant S., Hiyoshi H., Gotoh K., Iida T., Kodama T. (2019). Export of a *Vibrio parahaemolyticus* toxin by the Sec and type III secretion machineries in tandem. Nat. Microbiol..

[12] Stone C.B., Bulir D.C., Emdin C.A., Pirie R.M., Porfilio E.A., Slootstra J.W., Mahony J.B. (2011). *Chlamydia pneumoniae* CdsL regulates CdsN ATPase activity, and disruption with a peptide mimetic prevents bacterial invasion. Front. Microbiol..

[13] Nakamura K., Shinoda N., Hiramatsu Y., Ohnishi S., Kamitani S., Ogura Y., Hayashi T. (2019). Horiguchi YBspR/BtrA, an anti-σ, factor. Regulates the ability of *Bordetella bronchiseptica* to cause cough in rats. Msphere.

[14] Berube B.J., Murphy K.R., Torhan M.C., Bowlin N.O., Williams J.D., Bowlin T.L., Moir D.T., Hauser A.R. (2017). Impact of type III secretion effectors and of phenoxyacetamide inhibitors of type III Secretion on abscess formation in a mouse model of *Pseudomonas aeruginosa* infection. Antimicrob. Agents Chemother..

[15] Duncan M.C., Linington R.G., Auerbuch V. (2012). Chemical inhibitors of the type three secretion system: Disarming bacterial pathogens. Antimicrob. Agents Chemother..

[16] Kolár M., Urbánek K., Látal T. (2001). Antibiotic selective pressure and development of bacterial resistance. Int. J. Antimicrob. Agents.

[17] Kimura K., Iwatsuki M., Nagai T., Matsumoto A., Takahashi Y., Shiomi K., Omura S., Abe A. (2011). A small-molecule inhibitor of the bacterial type III secretion system protects against in vivo infection with *Citrobacter rodentium*. J. Antibiot..

[18] Loureiro I., Frankel G., Adu-Bobie J., Dougan G., Trabulsi L.R., Carneiro-Sampaio M.M.S. (1998). Human colostrum contains IgA antibodies reactive to enteropathogenic *Escherichia coli* virulence-associated proteins: Intimin, BfpA, EspA, and EspB. J. Pediatr. Gastroenterol. Nutr..

[19] Shimanovich A.A., Buskirk A.D., Heine S.J., Blackwelder W.C., Wahid R., Kotloff K.L., Pasetti M.F. (2017). Functional and antigen-specific serum antibody levels as correlates of protection against shigellosis in a controlled human challenge study. Clin. Vaccine Immunol..

[20] Gavilanes-Parra S., Mendoza-Hernández G., Chávez-Berrocal M.E., Girón J.A., Orozco-Hoyuela G., Manjarrez-Hernández A. (2013). Identification of secretory immunoglobulin A antibody targets from human milk in cultured cells infected with enteropathogenic *Escherichia coli* (EPEC). Microb. Pathog..

[21] Li Y., Frey E., Mackenzie A.M.R., Finlay B.B. (2000). Human response to *Escherichia coli* O157:H7 infection: Antibodies to secreted virulence factors. Infect. Immun..

[22] Durand D., Ochoa T.J., Bellomo S.M.E., Contreras C.A., Bustamante V.H., Ruiz J., Cleary T.G. (2013). Detection of secretory immunoglobulin a in human colostrum as mucosal immune response against proteins of the type III secretion system of *Salmonella*, *Shigella* and enteropathogenic *Escherichia coli*. Pediatr. Infect. Dis. J..

[23] Rabinovitz B.C., Gerhardt E., Tironi Farinati C., Abdala A., Galarza R., Vilte D.A., Ibarra C., Cataldi A., Mercado E.C. (2012). Vaccination of pregnant cows with EspA, EspB, γ-intimin, and Shiga toxin 2 proteins from *Escherichia coli* O157:H7 induces high levels of specific colostral antibodies that are transferred to newborn calves. J. Dairy Sci..

[24] Rabinovitz B.C., Vilte D.A., Larzábal M., Abdala A., Galarza R., Zotta E., Ibarra C., Mercado E.C., Cataldi A. (2014). Physiopathological effects of *Escherichia coli* O157: H7 inoculation in weaned calves fed with colostrum containing antibodies to EspB and Intimin. Vaccine.

[25] Mayne E., Prinz W., Van Dixhoorn M.S., Mayne E., Wadee A.A., Mendelow B., Ramsay M.N., Chetty W.S. (2009). Immunology. Molecular Medicine for Clinicians.

[26] Davies D.H., Morrow W.J.W., Sheikh N.A., Schmidt C.S., Davies H.D. (2012). Antigen Discovery for Vaccines Using High-throughput Proteomic Screening Techniques. Vaccinology: Principles and Practice.

[27] Li Y., Jin L., Chen T. (2020). The effects of secretory IgA in the mucosal immune system. BioMed Res. Int..

[28] Yanaka S., Yogo R., Kato K. (2020). Biophysical characterization of dynamic structures of immunoglobulin G. Biophys. Rev..

[29] Zurawski D.V., Mclendon M.K. (2020). Monoclonal antibodies as an antibacterial approach against bacterial pathogens. Antibiotics.

[30] Nagy E., Nagy G., Power C.A., Badarau A., Szijártó V. (2017). Anti-bacterial monoclonal antibodies. Adv. Exp. Med. Biol..

[31] Hu Y., Liu C., Muyldermans S. (2017). Nanobody-based delivery systems for diagnosis and targeted tumor therapy. Front. Immunol..

[32] Kolkman J.A., Law D.A. (2010). Nanobodies-From llamas to therapeutic proteins. Drug Discov. Today Technol..

[33] Forthal D.N. (2014). Functions of antibodies. Microbiol. Spectr..

[34] Sawa T., Kinoshita M., Inoue K., Ohara J., Moriyama K. (2019). Immunoglobulin for treating bacterial infections: One more mechanism of action. Antibodies.

[35] Goldberg B.S., Ackerman M.E. (2020). Antibody-mediated complement activation in pathology and protection. Immunol. Cell Biol..

[36] Abramov V.M., Kosarev I.V., Motin V.L., Khlebnikov V.S., Vasilenko R.N., Sakulin V.K., Machulin A.V., Uversky V.N., Karlyshev A.V. (2019). Binding of LcrV protein from *Yersinia pestis* to human T-cells induces apoptosis, which is completely blocked by specific antibodies. Int. J. Biol. Macromol..

[37] Jenner E. (1801). On the origin of the vaccine inoculation. Med. Phys. J..

[38] Davies D.H., Schmidt C.S., Sheikh N.A., Morrow W.J.W., Sheikh N.A., Schmidt C.S., Davies H.D. (2012). Concept and Scope of Modern Vaccines. Vaccinology: Principles and Practice.

[39] Frey S.E., Lottenbach K., Graham I., Anderson E., Bajwa K., May R.C., Mizel S.B., Graff A., Belshe R.B. (2017). A phase I safety and immunogenicity dose escalation trial of plague vaccine, Flagellin/F1/V, in healthy adult volunteers (DMID 08-0066). Vaccine.

[40] Boros P., Gondolesi G., Bromberg J.S. (2005). High dose intravenous immunoglobulin treatment: Mechanisms of action. Liver Transpl..

[41] Afonso A.F.B., João C.M.P. (2016). The production processes and biological effects of intravenous immunoglobulin. Biomolecules.

[42] Lee J.L., Mohamed Shah N., Makmor-Bakry M., Islahudin F.H., Alias H., Noh L.M., Mohd Saffian S. (2020). A systematic review and meta-regression analysis on the impact of increasing IgG trough level on infection rates in primary immunodeficiency patients on intravenous IgG therapy. J. Clin. Immunol..

[43] McCusker C., Warrington R. (2011). Primary immunodeficiency. Allergy Asthma Clin. Immunol..

[44] Chaigne B., Mouthon L. (2017). Mechanisms of action of intravenous immunoglobulin. Transfus. Apheresis Sci..

[45] Negi V.S., Elluru S., Sibéril S., Graff-Dubois S., Mouthon L., Kazatchkine M.D., Lacroix-Desmazes S., Bayry J., Kaveri S.V. (2007). Intravenous immunoglobulin: An update on the clinical use and mechanisms of action. J. Clin. Immunol..

[46] Ramus B., Benbrahim O., Chérin P. (2019). Use of intravenous and subcutaneous human immunoglobulins. Soins Rev. Ref. Infirm..

[47] Sriaroon P., Ballow M. (2015). Immunoglobulin replacement therapy for primary immunodeficiency. Immunol. Allergy Clin. N. Am..

[48] Aubron C., Berteau F., Sparrow R.L. (2019). Intravenous immunoglobulin for adjunctive treatment of severe infections in ICUs. Curr. Opin. Crit. Care.

[49] Kaplon H., Muralidharan M., Schneider Z., Reichert J.M. (2020). Antibodies to watch in 2020. MAbs.

[50] Sécher T., Dalonneau E., Ferreira M., Parent C., Azzopardi N., Paintaud G., Si-Tahar M., Heuzé-Vourc’h N. (2019). In a murine model of acute lung infection, airway administration of a therapeutic antibody confers greater protection than parenteral administration. J. Control Release.

[51] Ndungo E., Randall A., Hazen T.H., Kania D.A., Trappl-Kimmons K., Liang X., Barry E.M., Kotloff K.L., Chakraborty S., Mani S. (2018). A novel *Shigella* proteome microarray discriminates targets of human antibody reactivity following oral vaccination and experimental challenge. Msphere.

[52] Hill J., Eyles J.E., Elvin S.J., Healey G.D., Lukaszewski R.A., Titball R.W. (2006). Administration of antibody to the lung protects mice against pneumonic plague. Infect. Immun..

[53] Clemens J., Elyazeed R.A., Rao M., Savarino S., Morsy B.Z., Kim Y., Wierzba T., Naficy A., Lee Y.J. (1999). Early initiation of breastfeeding and the risk of infant diarrhea in rural Egypt. Pediatrics.

[54] Kapil P., Papin J.F., Wolf R.F., Zimmerman L.I., Wagner L.D., Merkel T.J. (2018). Maternal vaccination with a monocomponent pertussis toxoid vaccine is sufficient to protect infants in a baboon model of Whooping cough. J. Infect. Dis..

[55] Kaplon H., Reichert J.M. (2019). Antibodies to watch in 2019. MAbs.

[56] Gura T. (2002). Magic bullets hit the target. Nature.

[57] Hollowell P., Li Z., Hu X., Ruane S., Kalonia C., Van der Walle C.F., Lu J.R. (2020). Recent advances in studying interfacial adsorption of bioengineered monoclonal antibodies. Molecules.

[58] Buyel J.F., Twyman R.M., Fischer R. (2017). Very-large-scale production of antibodies in plants: The biologization of manufacturing. Biotechnol. Adv..

[59] Gómez-Mantilla J.D., Trocóniz I.F., Parra-Guillén Z., Garrido M.J. (2014). Review on modeling anti-antibody responses to monoclonal antibodies. J. Pharmacokinet. Pharmacodyn..

[60] Glück D., Wiedeck H., van Wickern M., Wölpl A., Northoff H., Ahnefeld F.W., Grünert A., Kubanek B. (1990). Anti-lipopolysaccharide-immunoglobulin (IgG-Anti-LPS) therapy in intensive care patients following surgery from infectious disease. Infusiontherapie..

[61] Shime N., Sawa T., Fujimoto J., Faure K., Allmond L.R., Karaca T., Swanson B.L., Spack E.G., Wiener-Kronish J.P. (2001). Therapeutic administration of anti-PcrV F(ab′)_2_ in sepsis associated with *Pseudomonas aeruginosa*. J. Immunol..

[62] Fasciano A.C., Shaban L., Mecsas J., Alyssa C. (2019). Fasciano1, Lamyaa Shaban2, and J.M.; Fasciano, A.C.; Shaban, L.; Mecsas, J. Promises and challenges of the type three secretion system- injectisome as an anti-virulence target. EcoSal Plus.

[63] Baron C., Coombes B. (2008). Targeting bacterial secretion systems: Benefits of disarmament in the microcosm. Infect. Disord. Drug Targets.

[64] Hilf M., Yu V.L., Sharp J., Zuravleff J.J., Korvick J.A., Muder R.R. (1989). Antibiotic therapy for *Pseudomonas aeruginosa* bacteremia: Outcome correlations in a prospective study of 200 patients. Am. J. Med..

[65] Secher T., Fas S., Fauconnier L., Mathieu M., Rutschi O., Ryffel B., Rudolf M. (2013). The anti-*Pseudomonas aeruginosa* antibody Panobacumab is efficacious on acute pneumonia in neutropenic mice and has additive effects with meropenem. PLoS ONE.

[66] Le H.N., Tran V.G., Vu T.T.T., Gras E., Le V.T.M., Pinheiro M.G., Aguiar-Alves F., Schneider-Smith E., Carter H.C., Sellman B.R. (2019). Treatment efficacy of MEDI3902 in *Pseudomonas aeruginosa* bloodstream infection and acute pneumonia rabbit models. Antimicrob. Agents Chemother..

[67] Kunert R., Reinhart D. (2016). Advances in recombinant antibody manufacturing. Appl. Microbiol. Biotechnol..

[68] Kang S., Ren D., Xiao G., Daris K., Buck L., Enyenihi A.A., Zubarev R., Bondarenko P.V., Deshpande R. (2014). Cell line profiling to improve monoclonal antibody production. Biotechnol. Bioeng..

[69] Huang Y.M., Hu W.W., Rustandi E., Chang K., Yusuf-Makagiansar H., Ryll T. (2010). Maximizing productivity of CHO cell-based fed-batch culture using chemically defined media conditions and typical manufacturing equipment. Biotechnol. Prog..

[70] Itoh Y., Ueda H., Suzuki E. (1995). Overexpression of bcl-2, apoptosis suppressing gene: Prolonged viable culture period of hybridoma and enhanced antibody production. Biotechnol. Bioeng..

[71] Nishimiya D., Mano T., Miyadai K., Yoshida H., Takahashi T. (2013). Overexpression of CHOP alone and in combination with chaperones is effective in improving antibody production in mammalian cells. Appl. Microbiol. Biotechnol..

[72] Sittner A., Mechaly A., Vitner E., Aftalion M., Levy Y., Levy H., Mamroud E., Fisher M. (2018). Improved production of monoclonal antibodies against the LcrV antigen of *Yersinia pestis* using FACS-aided hybridoma selection. J. Biol. Methods.

[73] Zhou Y., Liu P., Gan Y., Sandoval W., Katakam A.K., Reichelt M., Rangell L., Reilly D. (2016). Enhancing full-length antibody production by signal peptide engineering. Microb. Cell Fact..

[74] Saberianfar R., Chin-Fatt A., Scott A., Henry K.A., Topp E., Menassa R. (2019). Plant-produced chimeric V_H_H-sIgA against enterohemorrhagic *E. coli* intimin shows cross-serotype inhibition of bacterial adhesion to epithelial cells. Front. Plant Sci..

[75] Choi J.H., Lee S.Y. (2004). Secretory and extracellular production of recombinant proteins using *Escherichia coli*. Appl. Microbiol. Biotechnol..

[76] Kinoshita M., Shimizu M., Akiyama K., Kato H., Moriyama K., Sawa T. (2020). Epidemiological survey of serum titers from adults against various Gram-negative bacterial V-antigens. PLoS ONE.

[77] Moody A.M. (1920). The value of bacterial vaccines in immunization and therapy. J. Am. Med. Assoc..

[78] Meyer K.F., Cavanaugh D.C., Bartelloni P.J., Marshall J.D. (1974). Plague immunization. I. Past and present trends. J. Infect. Dis..

[79] Burrows T.W., Bacon G.A. (1958). The effects of loss of different virulence determinants on the virulence and immunogenicity of strains of *Pasteurella pestis*. Br. J. Exp. Pathol..

[80] Lawton W.D., Erdman R.L., Surgalla M.J. (1963). Biosynthesis and purification of V and W antigen in *Pasteurella pestis*. J. Immunol..

[81] Motin V.L., Nakajima R., Smirnov G.B., Brubaker R.R. (1994). Passive immunity to yersiniae mediated by anti-recombinant V antigen and protein A-V antigen fusion peptide. Infect. Immun..

[82] Perry R.D., Harmon P.A., Bowmer W.S., Straley S.C. (1986). A low-Ca2+ response operon encodes the V antigen of *Yersinia pestis*. Infect. Immun..

[83] Salmond G.P., Reeves P.J. (1993). Membrane traffic wardens and protein secretion in Gram-negative bacteria. Trends Biochem. Sci..

[84] Cowan C., Philipovskiy A.V., Wulff-Strobel C.R., Ye Z., Straley S.C. (2005). Anti-LcrV antibody inhibits delivery of Yops by *Yersinia pestis* KIM5 by directly promoting phagocytosis. Infect. Immun..

[85] Miller N.C., Quenee L.E., Elli D., Ciletti N.A., Schneewind O. (2012). Polymorphisms in the LcrV gene of *Yersinia enterocolitica* and their effect on plague protective immunity. Infect. Immun..

[86] Ivanov M.I., Hill J., Bliska J.B. (2014). Direct neutralization of type III effector translocation by the variable region of a monoclonal antibody to *Yersinia pestis* LcrV. Clin. Vaccine Immun..

[87] Xiao X., Zhu Z., Dankmeyer J.L., Wormald M.M., Fast R.L., Worsham P.L., Cote C.K., Amemiya K., Dimitrov D.S. (2010). Human anti-plague monoclonal antibodies protect mice from *Yersinia pestis* in a bubonic plague model. PLoS ONE.

[88] Van Blarcom T.J., Sofer-Podesta C., Ang J., Boyer J.L., Crystal R.G., Georgiou G. (2010). Affinity maturation of an anti-V antigen IgG expressed in situ through adenovirus gene delivery confers enhanced protection against *Yersinia pestis* challenge. Gene Ther..

[89] Zauberman A., Flashner Y., Levy Y., Vagima Y., Tidhar A., Cohen O., Bar-Haim E., Gur D., Aftalion M., Halperin G. (2013). YopP-expressing variant of *Y. pestis* activates a potent innate immune response affording cross-protection against yersiniosis and tularemia. PLoS ONE.

[90] Philipovskiy A.V., Cowan C., Wulff-Strobel C.R., Burnett S.H., Kerschen E.J., Cohen D.A., Kaplan A.M., Straley S.C. (2005). Antibody against V antigen prevents Yop-dependent growth of *Yersinia pestis*. Infect. Immun..

[91] Imamura Y., Yanagihara K., Fukuda Y., Kaneko Y., Seki M., Izumikawa K., Miyazaki Y., Hirakata Y., Sawa T., Wiener-Kronish J.P. (2007). Effect of anti-PcrV antibody in a murine chronic airway *Pseudomonas aeruginosa* infection model. Eur. Respir. J..

[92] Song Y., Baer M., Srinivasan R., Lima J., Yarranton G., Bebbington C., Lynch S.V. (2012). PcrV antibody-antibiotic combination improves survival in *Pseudomonas aeruginosa*-infected mice. Eur. J. Clin. Microbiol. Infect. Dis..

[93] Ranjbar M., Behrouz B., Norouzi F., Gargari S.L.M. (2019). Anti-PcrV IgY antibodies protect against *Pseudomonas aeruginosa* infection in both acute pneumonia and burn wound models. Mol. Immunol..

[94] Frank D.W., Vallis A., Wiener-Kronish J.P., Roy-Burman A., Spack E.G., Mullaney B.P., Megdoud M., Marks J.D., Fritz R., Sawa T. (2002). Generation and characterization of a protective monoclonal antibody to *Pseudomonas aeruginosa* PcrV. J. Infect. Dis..

[95] De Tavernier E., Detalle L., Morizzo E., Roobrouck A., De Taeye S., Rieger M., Verhaeghe T., Correia A., Van Hegelsom R., Figueirido R. (2016). High throughput combinatorial formatting of PcrV nanobodies for efficient potency improvement. J. Biol. Chem..

[96] Jain R., Beckett V.V., Konstan M.W., Accurso F.J., Burns J.L., Mayer-Hamblett N., Milla C., VanDevanter D.R., Chmiel J.F., Chmiel J.F. (2018). KB001-A, a novel anti-inflammatory, found to be safe and well-tolerated in cystic fibrosis patients infected with *Pseudomonas aeruginosa*. J. Cyst. Fibros..

[97] Warrener P., Varkey R., Bonnell J.C., DiGiandomenico A., Camara M., Cook K., Peng L., Zha J., Chowdury P., Sellman B. (2014). A novel anti-PcrV antibody providing enhanced protection against *Pseudomonas aeruginosa* in multiple animal infection models. Antimicrob. Agents Chemother..

[98] Sawa T., Ito E., Nguyen V.H., Haight M. (2014). Anti-PcrV antibody strategies against virulent *Pseudomonas aeruginosa*. Hum. Vaccine Immunother..

[99] Le H.N., Quetz J.S., Tran V.G., Le V.T.M., Aguiar-Alves F., Pinheiro M.G., Cheng L., Yu L., Sellman B.R., Stover C.K. (2018). MEDI3902 correlates of protection against severe *Pseudomonas aeruginosa* pneumonia in a rabbit acute pneumonia model. Antimicrob. Agents Chemother..

[100] Wang Q., Li H., Zhou J., Zhong M., Zhu D., Feng N., Liu F., Bai C., Song Y. (2014). PcrV antibody protects multi-drug resistant *Pseudomonas aeruginosa* induced acute lung injury. Respir. Physiol. Neurobiol..

[101] Lynch S.V., Flanagan J.L., Sawa T., Fang A., Baek M.S., Rubio-Mills A., Ajayi T., Yanagihara K., Hirakata Y., Kohno S. (2010). Polymorphisms in the *Pseudomonas aeruginosa* type III secretion protein, PcrV-Implications for anti-PcrV immunotherapy. Microb. Pathog..

[102] Warr G.W., Magor K.E., Higgins D.A. (1995). IgY: Clues to the origins of modern antibodies. Immunol. Today.

[103] Kinoshita M., Kato H., Yasumoto H., Shimizu M., Hamaoka S., Naito Y., Akiyama K., Moriyama K., Sawa T. (2016). The prophylactic effects of human IgG derived from sera containing high anti-PcrV titers against pneumonia-causing *Pseudomonas aeruginosa*. Hum. Vaccine Immunother..

[104] François B., Luyt C.E., Dugard A., Wolff M., Diehl J.L., Jaber S., Forel J.M., Garot D., Kipnis E., Mebazaa A. (2012). Safety and pharmacokinetics of an anti-PcrV PEGylated monoclonal antibody fragment in mechanically ventilated patients colonized with *Pseudomonas aeruginosa*: A randomized, double-blind, placebo-controlled trial. Crit. Care Med..

[105] Milla C.E., Chmiel J.F., Accurso F.J., Vandevanter D.R., Konstan M.W., Yarranton G., Geller D.E. (2014). Anti-PcrV antibody in cystic fibrosis: A novel approach targeting *Pseudomonas aeruginosa* airway infection. Pediatr. Pulmonol..

[106] Tabor D.E., Oganesyan V., Keller A.E., Yu L., McLaughlin R.E., Song E., Warrener P., Rosenthal K., Esser M., Qi Y. (2018). *Pseudomonas aeruginosa* PcrV and Psl, the molecular targets of bispecific antibody MEDI3902, are conserved among diverse global clinical isolates. J. Infect. Dis..

[107] Ali S.O., Yu X.Q., Robbie G.J., Wu Y., Shoemaker K., Yu L., DiGiandomenico A., Keller A.E., Anude C., Hernandez-Illas M. (2019). Phase 1 study of MEDI3902, an investigational anti-*Pseudomonas aeruginosa* PcrV and Psl bispecific human monoclonal antibody, in healthy adults. Clin. Microbiol. Infect..

[108] La Ragione R.M., Patel S., Maddison B., Woodward M.J., Best A., Whitelam G.C., Gough K.C. (2006). Recombinant anti-EspA antibodies block *Escherichia coli* O157:H7-induced attaching and effacing lesions in vitro. Microbes Infect..

[109] Girard F., Batisson I., Martinez G., Breton C., Harel J.J., Fairbrother J.M. (2006). Use of virulence factor-specific egg yolk-derived immunoglobulins as a promising alternative to antibiotics for prevention of attaching and effacing *Escherichia coli* infections. FEMS Immunol. Med. Microbiol..

[110] Cook S.R., Maiti P.K., DeVinney R., Allen-Vercoe E., Bach S.J., McAllister T.A. (2007). Avian- and mammalian-derived antibodies against adherence-associated proteins inhibit host cell colonization by *Escherichia coli* O157:H7. J. Appl. Microbiol..

[111] Yu S., Gu J., Wang H., Wang Q., Luo P., Wu C., Zhang W., Guo G., Tong W., Zou Q. (2010). Identification of a novel linear epitope on EspA from enterohemorrhagic *E. coli* using a neutralizing and protective monoclonal antibody. Clin. Immunol..

[112] Praekelt U., Reissbrodt R., Kresse A., Pavankumar A., Sankaran K., James R., Jesudason M., Anandan S., Prakasam A., Balaji V. (2014). Monoclonal antibodies against all known variants of EspA: Development of a simple diagnostic test for enteropathogenic *Escherichia coli* based on a key virulence factor. J. Med. Microbiol..

[113] Desin T.S., Mickael C.S., Lam P.K., Potter A.A., Köster W. (2010). Protection of epithelial cells from *Salmonella enterica* serovar Enteritidis invasion by antibodies against the SPI-1 type III secretion system. Can. J. Microbiol..

[114] Barta M.L., Guragain M., Adam P., Dickenson N.E., Patil M., Geisbrecht B.V., Picking W.L., Picking W.D. (2012). Identification of the bile salt binding site on IpaD from *Shigella flexneri* and the influence of ligand binding on IpaD structure. Proteins.

[115] Barta M.L., Shearer J.P., Arizmendi O., Tremblay J.M., Mehzabeen N., Zheng Q., Battaile K.P., Lovell S., Tzipori S., Picking W.D. (2017). Single-domain antibodies pinpoint potential targets within *Shigella* invasion plasmid antigen D of the needle tip complex for inhibition of type III secretion. J. Biol. Chem..

[116] Ivanov M.I., Noel B.L., Rampersaud R., Mena P., Benach J.L., Bliska J.B. (2008). Vaccination of mice with a Yop translocon complex elicits antibodies that are protective against infection with F1^−^
*Yersinia pestis*. Infect. Immun..

[117] Guirro M., de Souza R.L., Piazza R.M.F., Guth B.E.C. (2013). Antibodies to intimin and *Escherichia coli*-secreted proteins EspA and EspB in sera of Brazilian children with hemolytic uremic syndrome and healthy controls. Vet. Immunol. Immunopathol..

[118] Rabinovitz B.C., Larzábal M., Vilte D.A., Cataldi A., Mercado E.C. (2016). The intranasal vaccination of pregnant dams with Intimin and EspB confers protection in neonatal mice from *Escherichia coli* (EHEC) O157: H7 infection. Vaccine.

[119] Forbes S.J., Eschmann M., Mantis N.J. (2008). Inhibition of *Salmonella enterica* serovar Typhimurium motility and entry into epithelial cells by a protective antilipopolysaccharide monoclonal immunoglobulin A antibody. Infect. Immun..

[120] Forbes S.J., Martinelli D., Hsieh C., Ault J.G., Marko M., Mannella C.A., Mantis N.J. (2012). Association of a protective monoclonal IgA with the O antigen of *Salmonella enterica* serovar Typhimurium impacts type 3 secretion and outer membrane integrity. Infect. Immun..

[121] Goodin J.L., Raab R.W., McKown R.L., Coffman G.L., Powell B.S., Enama J.T., Ligon J.A., Andrews G.P. (2005). *Yersinia pestis* outer membrane type III secretion protein YscC: Expression, purification, characterization, and induction of specific antiserum. Protein Expr. Purif..

[122] Costa T.R., Felisberto-Rodrigues C., Meir A., Prevost M.S., Redzej A., Trokter M., Waksman G. (2015). Secretion systems in Gram-negative bacteria: Structural and mechanistic insights. Nat. Rev. Microbiol..

[123] Arnoldo A., Curak J., Kittanakom S., Chevelev I., Lee V.T., Sahebol-Amri M., Koscik B., Ljuma L., Roy P.J., Bedalov A. (2008). Identification of small molecule inhibitors of *Pseudomonas aeruginosa* exoenzyme S using a yeast phenotypic screen. PLoS Genet..

[124] Akopyan K., Edgren T., Wang-Edgren H., Rosqvist R., Fahlgren A., Wolf-Watz H. (2011). Translocation of surface-localized effectors in type III secretion. Proc. Natl. Acad. Sci. USA.

[125] Singh A.K., Kingston J.J., Murali H.S., Batra H.V. (2014). A recombinant bivalent fusion protein rVE confers active and passive protection against *Yersinia enterocolitica* infection in mice. Vaccine.

[126] González-Juarbe N., Shen H., Bergman M.A., Orihuela C.J., Dube P.H. (2017). YopE specific CD8+ T cells provide protection against systemic and mucosal *Yersinia pseudotuberculosis* infection. PLoS ONE.

[127] Kerschen E.J., Cohen D.A., Kaplan A.M., Straley S.C. (2004). The plague virulence protein YopM targets the innate immune response by causing a global depletion of NK cells. Infect. Immun..

[128] Rüter C., Silva M.R., Grabowski B., Lubos M.L., Scharnert J., Poceva M., von Tils D., Flieger A., Heesemann J., Bliska J.B. (2014). Rabbit monoclonal antibodies directed at the T3SS effector protein YopM identify human pathogenic *Yersinia* isolates. Int. J. Med. Microbiol..

[129] Pacheco A.R., Lazarus J.E., Sit B., Schmieder S., Lencer W.I., Blondel C.J., Doench J.G., Davis B.M., Waldor M.K. (2018). CRISPR screen reveals that EHEC’s T3SS and Shiga toxin rely on shared host factors for infection. MBio.

[130] Ruano-Gallego D., Yara D.A., Di Ianni L., Frankel G., Schüller S., Fernández L.Á. (2019). A nanobody targeting the translocated intimin receptor inhibits the attachment of enterohemorrhagic *E. coli* to human colonic mucosa. PLoS Pathog..

[131] Kühne S.A., Hawes W.S., La Ragione R.M., Woodward M.J., Whitelam G.C., Gough K.C. (2004). Isolation of recombinant antibodies against EspA and intimin of *Escherichia coli* O157:H7. J. Clin. Microbiol..

[132] Jones-Carson J., McCollister B.D., Clambey E.T., Vázquez-Torres A. (2007). Systemic CD8 T-cell memory response to a *Salmonella* pathogenicity island 2 effector is restricted to *Salmonella enterica* encountered in the gastrointestinal mucosa. Infect. Immun..

[133] Turbyfill K.R., Hartman A.B., Oaks E.V. (2000). Isolation and characterization of a *Shigella flexneri* invasion complex subunit vaccine. Infect. Immun..

[134] Desin T.S., Townsend H.G., Potter A.A. (2015). Antibodies directed against Shiga-toxin producing *Escherichia coli* serotype O103 type III secreted proteins block adherence of heterologous STEC serotypes to HEp-2 cells. PLoS ONE.

[135] Engel J., Balachandran P. (2009). Role of *Pseudomonas aeruginosa* type III effectors in disease. Curr. Opin. Microbiol..

[136] Corech R., Rao A., Laxova A., Moss J., Rock M.J., Li Z., Kosorok M.R., Splaingard M.L., Farrell P.M., Barbieri J.T. (2005). Early immune response to the components of the type III system *of Pseudomonas aeruginosa* in children with cystic fibrosis. J. Clin. Microbiol..

[137] Finco O., Frigimelica E., Buricchi F., Petracca R., Galli G., Faenzi E., Meoni E., Bonci A., Agnusdei M., Nardelli F. (2011). Approach to discover T- and B-cell antigens of intracellular pathogens applied to the design of *Chlamydia trachomatis* vaccines. Proc. Natl. Acad. Sci. USA.

[138] Tsai P.Y., Hsu M.C., Huang C.T., Li S.Y. (2007). Human antibody and antigen response to IncA antibody of *Chlamydia trachomatis*. Int. J. Immunopathol. Pharmacol..

[139] Freeman J.A., Rappl C., Kuhle V., Hensel M., Miller S.I. (2002). SpiC is required for translocation of *Salmonella* pathogenicity island 2 effectors and secretion of translocon proteins SseB and SseC. J. Bacteriol..

[140] Geng S., Qian S., Pan Z., Sun L., Chen X., Jiao X. (2015). Preparation of monoclonal antibodies against SpiC protein secreted by T3SS-2 of *Salmonella* spp.. Monoclon. Antib. Immunodiagn. Immunother..

[141] Singh K., Ejaz W., Dutta K., Thayumanavan S. (2019). Antibody delivery for intracellular targets: Emergent therapeutic potential. Bioconjugate Chem..

[142] Briers Y., Walmagh M., Van Puyenbroeck V., Cornelissen A., Cenens W., Aertsen A., Oliveira H., Azeredo J., Verween G., Pirnay J.P. (2014). Engineered endolysin-based “Artilysins” to combat multidrug-resistant Gram-negative pathogens. MBio.

[143] Alzogaray V., Danquah W., Aguirre A., Urrutia M., Berguer P., Véscovi E.G., Haag F., Koch-Nolte F., Goldbaum F.A. (2011). Single-domain llama antibodies as specific intracellular inhibitors of SpvB, the actin ADP-ribosylating toxin of *Salmonella* Typhimurium. FASEB J..

[144] Winstanley C., Hart C.A. (2001). Type III secretion systems and pathogenicity islands. J. Med. Microbiol..

[145] Zhang Y., Sun X., Qian Y., Yi H., Song K., Zhu H., Zonta F., Chen W., Ji Q., Miersch S. (2019). A potent Anti-SpuE antibody allosterically inhibits type III secretion system and attenuates virulence of *Pseudomonas aeruginosa*. J. Mol. Biol..

[146] Wang J., Wang J., Zhang L.H. (2020). Immunological blocking of spermidine-mediated host–pathogen communication provides effective control against *Pseudomonas aeruginosa* infection. Microb. Biotechnol..

